# Factors Related to Diet Quality: A Cross-Sectional Study of 1055 University Students

**DOI:** 10.3390/nu13103512

**Published:** 2021-10-05

**Authors:** Enrique Ramón-Arbués, José-Manuel Granada-López, Blanca Martínez-Abadía, Emmanuel Echániz-Serrano, Isabel Antón-Solanas, Benjamin Adam Jerue

**Affiliations:** 1 Faculty of Health Sciences, Campus Universitario Villanueva de Gállego, Universidad San Jorge, Villanueva de Gállego, 50830 Zaragoza, Spain; eramon@usj.es; 2 Research Group Transfercult (H27_20D), University of Zaragoza, 50009 Zaragoza, Spain; jmgranada@unizar.es (J.-M.G.-L.); eechaniz@unizar.es (E.E.-S.); 3 Department of Physiatrics and Nursing, Faculty of Health Sciences, University of Zaragoza, C/Domingo Miral S/N, 50009 Zaragoza, Spain; 4 Research Group Safety and Care (GIISA021), Institute of Research of Aragón, 50009 Zaragoza, Spain; 5 Occupational Health and Prevention Service, Zaragoza City Council, P° de La Mina 9, 50001 Zaragoza, Spain; bmartinez@zaragoza.es; 6 Research Group Nursing Research in Primary Care in Aragón (GENIAPA) (GIIS094), Institute of Research of Aragón, 50009 Zaragoza, Spain; 7 Faculty of Communication and Social Sciences, Campus Universitario Villanueva de Gállego, Universidad San Jorge, Villanueva de Gállego, 50830 Zaragoza, Spain; bajerue@usj.es

**Keywords:** diet quality, dietary guidelines, university students, cross-sectional study

## Abstract

Given that there is only a limited body of evidence available concerning the dietary habits of Spanish university students, the present study assesses the quality of this group’s diet, their adherence to the National Food-Based Dietary Guidelines, and the predictive factors of their diet quality. To do so, a cross-sectional study was performed on a sample of 1055 students. The quality of the participants’ diets was then analysed by using the Spanish Healthy Eating Index, and then their level of compliance was assessed in light of the dietary recommendations put forth by the Spanish Society for Community Nutrition. According to these standards, only 17.4% of the participants had a healthy diet. The level of compliance with the recommendations was poor, highlighting especially the low levels of “fruit” and “vegetables” that they consumed as well as high levels of “cold meats and cuts” and “sweets”. The factors that predicted a worse diet are being male, living alone, low levels of physical activity, smoking, high alcohol intake, leading a sedentary lifestyle, psychological distress, and insomnia (*p* < 0.005). Furthermore, participants with low or high body weights showed signs of a higher quality diet (*p* < 0.001). The present findings suggest that a significant proportion of university students ought to change their dietary habits; these also attest to the importance of developing strategies that are directly targeted at university students in order to promote a healthy diet.

## 1. Introduction

Research has repeatedly shown the important role that diet plays in maintaining one’s short and long-term health as well as its relation to life expectancy [1,2]. Accordingly, increasing diet quality could be independently associated with a reduced risk of death (by all causes) of up to 28% [3,4]. There is also evidence [5,6] that links the consumption of certain foods with the increased or decreased likelihood of suffering from certain diseases. All of these findings on the importance of diet have been taken into account by various organisations that have published dietary guidelines [7]. The level of compliance with these recommendations has in turn served as the basis for constructing indices that seek to assess the diet quality (DQ) of various groups and populations. Not only have these indices proven to be useful tools for assessing alimentary practices (e.g., diet diversity, moderation, etc.), but their use is also recommended for studies on the DQ of the general population as well as more targeted groups [8]. Internationally, these indices have been used to study the DQ of a range of groups [9,10,11,12], including university students [13,14]. Students, in particular, are an important target audience for public health actions, seeing that entering university constitutes a drastic change in lifestyle that can bring about new challenges and meaningfully affect the students’ habits and health. Rivalry between classmates, the pressure to succeed academically, changes in workload and support networks, new types of relationships, and, in some instances, moving away from home are all factors that can trigger new, risky behaviours that may compromise the future health of university students [15,16]. These young adults find themselves in a crucial moment in terms of acquiring and reinforcing many habits that impact health. When it comes to dietary habits, university is the time when previously learned patterns can be cemented, or new patterns can be learned and replace old ones [17,18]. Many variables can exert influence on the ways that university students nourish themselves, including individual factors (e.g., the lack of self-discipline or time constraints), support networks (e.g., the influence of peers or the lack of parental monitoring), the local environment (e.g., accessibility or the appeal and price of certain food products) as well as the macro environment (e.g., advertising) [19,20]. Other sociodemographic variables have also been connected to the DQ of university students, including the following: living alone [21], gender [22], satisfaction with one’s studies and academic performance [23], lack of information [24] as well as anxiety and depression [25].

In Spain, there have been numerous studies on the DQ of the general population [7,26,27,28] and even clinical populations [29,30]. That said, more targeted work on the DQ of young people and university students in particular has been scarcer, and the studies that do exist have generally relied on small samples and somewhat indirect approximations of DQ based on the adherence to the Mediterranean diet. Accordingly, there is an important gap in the research that needs to be filled, especially given that universities can easily communicate with large groups of young adults and hence could become excellent agents for promoting healthier dietary practices. With this in mind, this study’s objectives were twofold: first, to assess Spanish university students’ DQ and adherence to the National Food-Based Dietary Guidelines (NFBDG) [31]; second, to identify possibly linked factors. 

## 2. Materials and Methods

### 2.1. Design and Study Population

**Figure 1:**
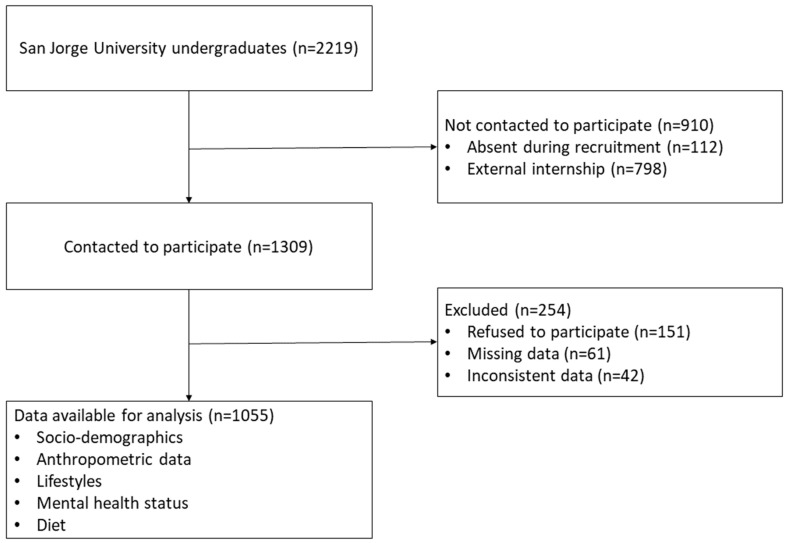
Study flow chart.

A descriptive cross-sectional study was carried out among a group of undergraduate students from the San Jorge University in Zaragoza (Aragon, Spain). Information about the research objectives was provided and students were recruited in the classroom during the second semester of the 2020–2021 academic year (specifically, in March and April of 2021). A total of 1309 students out of the 2219 enrolled at the university were asked to participate in the study by anonymously filling out a series of questionnaires. There were 151 students who declined to participate, while the responses of an additional 103 students were discarded due to gaps in the provided information (Figure 1).

### 2.2. Data Collection

Sociodemographic data (age, gender, place of residence, and marital status), anthropometric measurements (height and weight, which were used to calculate BMI), information about mental health history (signs of depression, anxiety, and stress), and lifestyle (current tobacco and alcohol intake, sleep, level of physical activity, time spent sitting, and diet) were collected. The BMI variable was categorised according to the classification of the World Health Organization (WHO) [32], namely as underweight (<18.5 kg/m^2^), normal weight (18.5–24.9 kg/m^2^), overweight (25–29.9 kg/m^2^), and obese (≥30 kg/m^2^). However, we combined the participants who were either overweight or obese into one group—overweight/obese (≥25 kg/m^2^)—in order to increase the sample-size comparability of the groups. The sociodemographic, anthropometric, and smoking data were all self-reported using a questionnaire made specifically for collecting this information. 

Data on the mental health of participating students were gathered through the Depression, Anxiety, Stress Scales-21 (DASS-21) [33]. This scale is an abridged version of the DASS-42 and was validated for the study of Spanish university students in 2010 [34]. DASS-21 consists of three sub-scales (depression, anxiety, and stress) with seven Likert-style questions (with choices between 0 and 3) in each section. The total score of each sub-scale is multiplied by two so that results can be compared with those from the DASS-42. Next, the subjects were classified according to the following criteria:

- Anxiety: Normal (0–7 points), mild (8–9), moderate (10–14), severe (15–19), and extremely severe (>19);
- Depression: Normal (0–9 points), mild (10–13), moderate (14–20), severe (21–27), and extremely severe (>27);
- Stress: Normal (0–14 points), mild (15–18), moderate (19–25), severe (26–33), and extremely severe (>33).

Data on alcohol intake were collected through the CAGE questionnaire, which has been validated for studying Spaniards by Rodríguez Martos et al. [35]. This questionnaire consists of four items with two possible responses (Yes or No). Consumption is deemed problematic when two or more questions are answered affirmatively.

Sleep quality was assessed using the Insomnia Severity Index (ISI) in its Spanish version [36]. This tool has previously been used to measure this construct in similar populations [37,38]. In its Spanish version, the ISI comprises 7 items measuring three different components of insomnia, namely (1) nature of insomnia, (2) severity of insomnia symptoms, and (3) impact of insomnia on daily function. Each item is rated on a 5-point Likert scale ranging from 0 to 4 points. The global score, ranging from 0 to 28, is obtained by adding the scores from each individual item. The results are classified as follows: (1) no insomnia (0–7 points), (2) sub-threshold insomnia (8–14 points), (3) moderate insomnia (15–21 points), severe insomnia (22–28 points).

Physical activity and sedentary time were assessed using the short version of the International Physical Activity Questionnaire (IPAQ-SF). This research tool, which has been validated for the study of Spanish university students [39], measures the intensity, frequency, and duration of physical activity over the last seven days. IPAQ-SF determines a mean of daily sedentary time and allows researchers to obtain two types of data concerning physical activity: first, it offers a calculation of the metabolic equivalent of tasks (METs), taking the type of activity (walking, moderate physical activity, and vigorous physical activity) and the time spent on the activity into account; secondly, the tool allows researchers to classify individuals into three categories of physical activity (low, moderate, high) [40].

DQ was assessed using the Spanish Healthy Eating Index (SHEI) [26]. This tool comprises 10 items measured on a 5-point Likert scale. Points for each answer (0 = no adherence, 2.5, 5, 7.5, or 10 = full adherence) are given based on the participant’s degree of adherence to the NFBDG recommendations for the frequency of consumption of specific foods [31]. Specifically, the SHEI measures the frequency of consumption of bread and grains, vegetables, fruit, dairy products, meat (including eggs), legumes, cold meats and cuts, sweets, soft drinks with sugar, and diet variety. The diet variety variable is calculated a posteriori by the researcher based on the participant’s report of daily and weekly food consumption (1 point is awarded for each weekly recommendation and 2 points for each daily recommendation that is fully met or adhered to). Supplementary Table S1 details the specific criteria for scoring each of these categories. The final score falls between 0 and 100 points, which is calculated by adding the scores from each category. Based on the results, diet can be classified as follows: healthy (>80), needing change (51–80), and inadequate (<51) [26].

### 2.3. Data Analysis

The characteristics of the sample were summarised using mean and standard deviation for the continuous variables, and frequency and percentage for the nominal ones. The Kolmogorov–Smirnov test was used to check the normality of DQ (SHEI score). Next, a multiple linear regression analysis was carried out (enter method) to determine which factors were associated with DQ (SHEI score). The covariates included in the multiple linear regression model were age, gender, BMI (WHO categories), life arrangement, marital status, smoking status, alcohol consumption (CAGE categories), physical activity (IPAQ categories), sedentary time, sleep quality (ISI categories), and symptoms of depression, anxiety, and stress (DASS-21 categories).

The model’s goodness-of-fit was assessed through R^2^. Furthermore, the diagnostic of collinearity of the final regression model showed tolerance levels over 0.6 for all included variables. The statistical analysis of the data was performed with the SPSS statistical package for Windows-Version 21 (IBM Corp, Armonk, NY, USA), accepting a significance level of *p* < 0.05.

## 3. Results

The size of the study’s final sample came to 1055 university students, with an average age of 21.74 ± 5.15. The majority of the participants were women (70.5%), living with family (66.4%), and non-smokers (76.6%). Nearly a third reported high alcohol intake and nearly half reported a low level of physical activity. Furthermore, 33.9% of the students reported some degree of stress, 18.5% reported depression, 23.5% anxiety, and 43.1% insomnia. Other information about the sample is presented in Table 1.

The average SHEI score came up to 68.57 ± 12.17 (100 being the maximum). The vast majority of the participants reported an inadequate diet (6.9%) or a diet needing changes (75.6%), while only 17.4% had a healthy diet (Table 1). The scores obtained by the participants ranged between 4.45 ± 3.28, for the consumption of sweets, and 9.27 ± 2.82, for the consumption of dairy products (Table 2).

**Figure 2:**
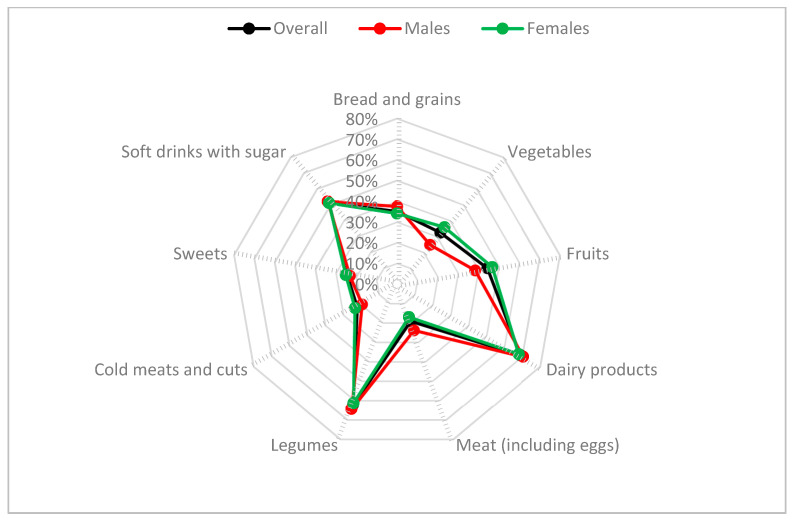
Percentage of students who adhere to the NFBDG-2016 [31] recommendations for the frequency with which certain foods should be consumed every week.

Most participants showed a low level of compliance with the NFBDG recommendations concerning the frequency with which certain foods are consumed, especially for “bread and grains”, “fruits”, “meat”, “vegetables”; in contrast, participants surpassed recommendations for the categories “cold meats and cuts” and “sweets” (Figure 2). Men and women reported similar dietary patterns; that said, the women more consistently followed the recommendations for the consumption of fruits and vegetables (*p* < 0.05), whereas the men more rigidly adhered to recommendations concerning meat (*p* < 0.05).

Through the multiple linear regression analysis, we found that being older, being female, not having a stable partner as well as having a low or high body weight independently correlated with a better DQ (*p* < 0.05). On the other hand, demonstrating signs of depression, anxiety, stress or insomnia, living alone, smoking, having an unactive lifestyle, being sedentary, and a high alcohol intake were all linked independently with lower SHEI scores (*p* < 0.05). This model’s predictive power for DQ was determined to be 37.7% (R^2^ = 0.38) (Table 3).

## 4. Discussion

Based on the recommendations of the National Food-Based Dietary Guidelines [31], our findings suggest that university students’ diet quality is generally poor. Our participants consumed insufficient amounts of “fruit” and “vegetables” and excessive amounts of “cold meats and cuts” and “sweets”. Factors that predicted a diet of worse quality were being male, living alone, maintaining low levels of physical activity, smoking, consuming alcohol, leading a sedentary lifestyle, experiencing psychological distress, and insomnia. In contrast, participants with low or high body weights showed signs of a higher quality diet.

Only 17.4% of the participants reported having a healthy diet. The present results are similar to those previously obtained by using SHEI to study Spanish university students [22,41,42,43]. They are, however, distinctly lower than the results from studies of the general Spanish population, for which between 28% and 35.8% of the participants reported having a healthy diet [26,27,29]. These results could be explained in two ways that are not mutually exclusive. First, the newly found liberty and independence associated with university life could allow students to let themselves go and overindulge in low-quality foods and alcohol. This does not necessarily mean that these individuals will not improve their DQ in the future after finishing their studies; indeed, they very well could do so. In this vein, previous studies carried out in England, Spain, and Lebanon have shown that some students opt for a poorer diet and gain a significant amount of weight in the first year of university, a phenomenon popularly referred to as “the Freshman 15” [44,45,46]. The second argument is that these trends reflect a more general tendency and could therefore imply that the DQ of Spaniards could decline in the future. The fact that age was positively associated with DQ in our sample lends support to this hypothesis. Indeed, research has shown that there has continually been less adherence to the Mediterranean diet in Spain [47,48], a diet that has been shown to be healthy and traditional in the country. This, however, does not appear to be a trend that is only valid in Spain, since researchers throughout Europe have detected a decrease in DQ over the last decade [49]. 

Internationally, researchers have found a remarkable range in the percentage of university students who have a healthy diet, ranging between 1.8% and 57% [18,50,51,52,53]. These differences, for the most part, can be explained in terms of the different socioeconomic realities and living arrangements of the various countries studied. For example, whereas 57% of Thai students reported following an inadequate diet [50], only 2.03% of Northern European students followed an unhealthy diet [18].

What deserves special attention is how student habits do not conform to the national alimentary guidelines for the majority of food groups. In fact, over 50% of the participants complied with recommendations in only three of the 10 variables included in the SHEI (dairy products, legumes, and soft drinks with sugar). Likewise, the results show a low level of fruit and vegetable consumption (44.3% and 32.5% of recommended frequency, respectively). Prima facie, these results may come as a surprise since Spain has a reputation for the quality of its agricultural products. However, similar trends have already been observed in Spanish university students [54,55] and the general population [7,56] that support the present findings. 

In line with previous studies carried out in Tunisia and Canada [52,57], being a woman is associated with a better DQ. The relationship between gender and diet are conditioned by a suite of physiological, psychological, and sociocultural factors. Thus, women generally believe in the importance of a healthy diet, are more likely to monitor their body weight, and frequently express greater concern over their own eating habits [58,59,60].

The connection between DQ and BMI is controversial. From a biological point of view, it is plausible that a low-quality diet could be associated with deviations (whether higher or lower) from what experts recommend as a healthy body weight. As a corollary, a high-quality diet would be expected to favour healthy body weight. However, various studies have demonstrated how this is not always the case [61,62]. Our analysis found a U-shaped relation between the profiles of BMI and DQ (healthy body weight < low and high weights). These results should be viewed with caution given this study’s qualitative means of assessing DQ. However, it is possible that in a developed country such as Spain, young university students who are over or underweight may be more informed and conscious about what constitutes a healthy and balanced diet; perhaps some are influenced by the enduring canon of beauty in the West (based on being thin), while others seek to have a healthier weight.

The participants that lived alone showed a poorer DQ than those who lived with family members or flat mates. This same phenomenon has been repeatedly found in other studies involving samples of European students [21,63]. There are various reasons that explain why students who live alone were less likely to adopt a healthy diet; these include, but are not limited to, the following: changing lifestyle, the comfort and convenience of fast food, taste, students’ physical and social environment, and awareness of weight [64].

The fact that being in a stable relationship was associated with a poorer DQ in our sample appears to support the prevalent idea that when we enter a serious relationship, we often do not take as good care of ourselves as before. Available research on the issue has shown that people who are overweight or obese (especially women) have more difficulty finding a partner than those who are not overweight [65]. Developing this idea, van Woerden et al. [66] suggest that there is a selection bias related to weight for beginning (but not ending) romantic relationships. In their study, these authors observed that for students that were single at moment A, the increase of each BMI unit reduced their chances of finding a partner at moment B (four months later) by 9%. Taking these results into account, one could infer that an individual seeking to start a new relationship might be more inclined to regulate his/her diet than someone uninterested in finding a new partner.

Among the study population, a clear association was observed between DQ and both unhealthy lifestyle choices (i.e., low levels of physical activity, a highly sedentary lifestyle, high alcohol intake, or smoking) as well as psychological health (i.e., the presence of stress, anxiety, depression, or insomnia). Previous studies have shown the tendency for unhealthy lifestyle choices (including low-quality diet) to cluster among Spanish university students [67,68,69,70]. The same holds for the relation between mental health and DQ [22,23,41]. Unhealthy habits (e.g., high alcohol intake, smoking, or certain dietary behaviours) have often been detected among those suffering from psychological distress and have been identified as passive coping mechanisms based on avoidance and/or escape [71]. However, one should not dismiss a bidirectional relationship between mental health and certain unhealthy lifestyle choices such as poor DQ. In fact, several recent studies have reported a strong correlation between a healthy diet and psychological wellbeing. Therefore, healthy diets such as the Mediterranean, which is rich in fresh fruits and vegetables, could be associated with higher levels of happiness and mental health [72,73,74], whereas certain gaps in the diet could be associated with a deterioration in mental health [71,75]. A similar connection can be observed when it comes to sleep quality; participants suffering from insomnia also reported a worse DQ. These results are consistent with findings in the broader scholarly literature on the topic. Numerous experimental studies have shown how partial sleep deprivation leads to an increased intake of fats [76,77], snacks [78,79], foods rich in rapidly absorbed carbohydrates [80,81], and inconsistent or shifting mealtimes [76]. Similarly, observational studies have provided further evidence of poorer DQ among people who have a lower quality of sleep in Iran and the USA [82,83]. Different explanations have been proffered to illuminate the phenomenon. More waking hours means more time for eating, changes in the level of hormones that regulate appetite (leptin and ghrelin), increased processing of hedonic stimuli in the brain (providing a greater reward from food), and shifting eating schedules [84]. However, it may not only be lack of sleep that affects diet; certain foods and nutrients can themselves influence sleep quality. For example, high-carb diets and foods that contain tryptophan, melatonin, and phytonutrients (e.g., cherries) seem to be linked with higher sleep quality [85]. 

As far as the authors are aware, this is the first study that analyses DQ and a large number of sociodemographic and behavioural variables among a large sample of Spanish university students. We believe that the obtained results can be generalised to the larger body of Spanish university students, not only due to the large sample size but also because of the standardised procedures used to collect data as well as the plausibility of the associations detected in the data analysis. Accordingly, this would allow us to trace a reliable picture of university students’ DQ as well as its associated factors. Such information is crucial since it can assist in the development and implementation of both diagnostic tools as well as educational activities concerning a healthy diet. That said, the study also has several limitations that need to be underscored. First, the transversal design makes it possible to detect associations, but it does not allow us to determine a cause-and-effect relationship or direction of influence. Second, the type of sampling used, a choice based on convenience and available resources, does impinge on our ability to draw generalisations from these results even though, we should note, the profile of our sample (i.e., predominantly female) does match the broader demographics of university students in Aragon. Third, data was self-reported by the participants, which means that one cannot rule out the possibility of faulty memory or the playing down of certain information such as body weight [86,87]. Fourth, our data was collected when the COVID-19 pandemic and the resulting public health measures were well underway in Spain. This fact provides valuable information about Spanish university students’ diet in the current context, and, in all likelihood, our results reflect the pandemic’s impact on the lives and dietary habits of university students. However, the data does not allow us to determine the precise ways that the current situation has affected students’ diets. Nevertheless, it is worth mentioning that other researchers have observed a decline in DQ since the beginning of the pandemic; lockdown measures have, on the one hand, led to a decline in the consumption of fruits and vegetables [88], and on the other hand, to an increase in the consumption of unhealthy foods (e.g., highly-processed foods, snacks, and frozen foods) [89,90,91]. Furthermore, the SHEI questionnaire only takes into account the frequency with which foods are consumed and not the quantity that is ingested. For this reason, it cannot offer information concerning the intake of nutrients or calories. Despite these limitations, there were three main reasons that we chose this tool: it is a questionnaire specifically adapted to Spanish alimentary habits; it has repeatedly been shown to be well-equipped to assess the DQ of Spaniards [27,92,93]; finally, the original questionnaire from which it was derived has been validated with plasma biomarkers in previous studies [94,95]. Given the limitations listed above, further research on the present issue is needed, including a longitudinal study as well as a quantitative approach to evaluating the diet of Spanish university students.

## 5. Conclusions and Recommendations

The present findings reveal that a large portion of university students have a poor DQ and do not closely adhere to alimentary recommendations. Therefore, it is necessary to encourage changes in current dietary patterns. Factors such as living arrangement, certain unhealthy habits (high alcohol intake, a sedentary lifestyle, smoking, and the presence of psychological distress and insomnia) predict DQ and can therefore be used to help tailor and direct action. It is indeed essential that educational institutions and health services undertake future action to promote better DQ among university students. We recommend a global approach to the issue that takes into account the factors related to poor DQ. Any such actions ought to take into consideration the following recommendations:

(1) the early detection of trends of poor DQ among students entering university as part of existing systems that offer students support;
(2) activities that aim to interrupt and prevent unhealthy dietary habits such as providing gender-specific information about immediate health concerns or organising community-wide public health campaigns that ensure that students have access to needed resources and affordable healthy foods;
(3) the empowerment of students by encouraging, for example, the acquisition of skills for making dietary choices and increasing resilience so that they can develop their own adaptive strategies to confront dietary problems and avoid unhealthy dietary habits;
(4) the consideration and handling of any underlying mental health conditions that could be present among university students with poor DQ.

## Figures and Tables

**Table 1:** Participant characteristics (*n* = 1055).

Variable		Mean ± SD/*n* (%)
Age		21.74 ± 5.15
Gender	Female	744 (70.5%)
	Male	311 (29.5%)
BMI (kg/m^2^)		22.15 ± 3.48
BMI Categories	Underweight (<18.5 kg/m^2^)	160 (15.2%)
	Normal weight (18.5–24.9 kg/m^2^)	736 (69.8%)
	Overweight/obese (≥25 kg/m^2^)	159 (15.1%)
Living arrangement	Living alone	64 (6.1%)
	Living with a partner	291 (27.6%)
	Living with family	700 (66.4%)
Relationship status	Currently in a relationship	494 (46.8%)
	Currently not in a relationship	561 (53.2%)
Smoking status (current)	No	535 (76.6%)
	Yes	163 (23.4%)
CAGE score		0.48 ± 0.78
CAGE categories	Problematic alcohol consumption	343 (32.5%)
	Non-problematic alcohol consumption	712 (67.5%)
SHEI score		68.57 ± 12.17
Diet quality	Inadequate (<51 points)	73 (6.9%)
	Need changes (51–80 points)	798 (75.6%)
	Healthy (>80 points)	184 (17.4%)
Physical activity (METs/week)		1877.03 ± 1966.54
Physical activity categories	High	236 (22.4%)
	Medium	351 (33.3%)
	Low	468 (44.4%)
Sedentary time (hours/day)		6.76 ± 2.45
Sedentary time categories	<3 h/day	133 (12.6%)
	3–6 h/day	342 (32.4%)
	≥6 h/day	580 (54.9%)
DASS-E score		12.39 ± 8.08
DASS-E categories	No stress	697 (66.1%)
	Mild stress	121 (11.5%)
	Moderate stress	174 (16.5%)
	Severe stress	46 (4.4%)
	Extremely severe stress	17 (1.6%)
DASS-D score		5.45 ± 7.12
DASS-D categories	No depression	859 (81.4%)
	Mild depression	80 (7.6%)
	Moderate depression	48 (4.5%)
	Severe depression	38 (3.6%)
	Extremely severe depression	30 (2.8%)
DASS-A score		4.84 ± 5.75
DASS-A categories	No anxiety	807 (76.5%)
	Mild anxiety	83 (7.9%)
	Moderate anxiety	95 (9.0%)
	Severe anxiety	9 (0.9%)
	Extremely severe anxiety	61 (5.8%)
ISI score		7.91 ± 4.88
Sleep quality	No insomnia	600 (56.9%)
	Sub-threshold insomnia	333 (31.6%)
	Moderate insomnia	114 (10.8%)
	Severe insomnia	8 (0.8%)

**Table 2:** SHEI scores of the participants (*n* = 1055).

Food Group	Mean ± SD	Min.	Max.	Mode
Bread and grains	7.27 ± 3.25	0	10	10 (Consumed daily)
Vegetables	8.38 ± 2.91	0	10	10 (Consumed daily)
Fruits	8.23 ± 3.54	0	10	10 (Consumed daily)
Dairy products	9.27 ± 2.82	0	10	10 (Consumed daily)
Meat	6.90 ± 2.83	0	10	7.5 (3 or more times a week but not daily)
Legumes	9.17 ± 1.97	0	10	7.5 (3 or more times a week but not daily)
Cold meats and cuts	6.29 ± 3.75	0	10	5 (Once or twice a week)
Sweets	4.45 ± 3.28	0	10	5 (Once or twice a week)
Soft drinks with sugar	8.62 ± 3.36	0	10	7.5 (Less than once a week)
SHEI score	68.57 ± 12.17	25	96.5	70.5 (Need changes)

**Table 3:** Factors related to diet quality. Multiple linear regression model *.

Independent Variable	B (CI 95%)	Std. Error	β	*p*
Age. <20 years (Ref.)				
20–24.9	3.54 (2.05, 5.04)	0.76	0.14	0.00
≥25	4.76 (2.68, 6.85)	1.06	0.14	0.00
Gender. Male (Ref.)				
Female	2.47 (0.94, 4.00)	0.781	0.09	0.00
BMI categories. Normal weight (Ref.)				
Underweight (<18.5 kg/m^2^)	3.55 (1.63, 5.48)	0.98	0.10	0.00
Overweight/obese (≥25 kg/m^2^)	6.42 (4.53, 8.31)	0.96	0.19	0.00
Living arrangement. Living with family (Ref.)				
Living alone	−11.49 (−14.27, −8.71)	1.42	−0.22	0.00
Living with a partner	−0.50 (−2.06, 1.07)	0.80	−0.02	0.53
Relationship status. Currently in a relationship (Ref.)				
Currently not in a relationship	3.63 (2.01, 5.25)	0.83	0.15	0.00
Smoking status. Not a smoker (Ref.)				
Smoker	−2.82 (−4.41, −1.24)	0.81	−0.10	0.00
CAGE Categories. Non-problematic alcohol consumption (Ref.)				
Problematic alcohol consumption	−1.82 (−3.20, −0.45)	0.702	−0.070	0.01
Physical activity. Medium (Ref.)				
High PA	1.74 (−0.05, 3.53)	0.91	0.06	0.06
Low PA	−2.77 (−4.45, −1.09)	0.86	−0.11	0.00
Sedentary time. 3–6 h per day				
<3 h/day	−0.29 (−2.48, 1.90)	1.11	−0.01	0.80
≥6 h/day	−2.40 (−3.86, −0.93)	0.75	−0.10	0.00
DASS-E categories. No stress (Ref.)				
Mild stress	12.10 (9.63, 14.57)	1.26	0.32	0.00
Moderate stress	−1.08 (−3.08, 0.92)	1.02	−0.03	0.29
Severe stress	−0.29 (−3.93, 3.35)	1.85	−0.00	0.88
Extremely severe stress	−5.56 (−11.37, 0.24)	2.96	−0.06	0.06
DASS-D categories. No depression (Ref.)				
Mild depression	2.13 (−1.00, 5.27)	1.60	0.05	0.18
Moderate depression	−3.23 (−6.71, 0.25)	1.77	−0.05	0.07
Severe depression	−18.04 (−22.12, −3.97)	2.07	−0.28	0.00
Extremely severe depression	−7.77 (−12.22, −3.33)	2.26	−0.11	0.00
DASS-A categories. No anxiety (Ref.)				
Mild anxiety	−3.25 (−5.91, −0.60)	1.35	−0.07	0.01
Moderate anxiety	−3.32 (−5.93, −0.70)	1.33	−0.08	0.01
Severe anxiety	−10.96 (−18.25, −3.66)	3.72	−0.08	0.00
Extremely severe anxiety	−1.29 (−5.18, 2.61)	1.98	−0.02	0.52
ISI categories. No insomnia (Ref.)				
Sub-threshold insomnia	−4.93 (−6.50, −3.37)	0.79	−0.19	0.00
Moderate insomnia	−6.62 (−9.11, −4.14)	1.27	−0.17	0.00
Severe insomnia	−13.51 (−21.46, −5.56)	4.05	−0.10	0.00

Ref. = Group of Reference. * Model’s Goodness of Fit (R^2^) = 0.38. *p* value (model) = 0.00 (one-way ANOVA).

## Data Availability

The data presented in this study are available on request from the corresponding author. The data are not publicly available as they contain information that could compromise the privacy of research participants.

## Supplementary Materials

The following are available online at https://www.mdpi.com/article/10.3390/nu13103512/s1, Table S1: Criteria for scoring the Spanish Healthy Eating Index (SHEI).
