# Supplementary material for: Factors Related to Diet Quality: A Cross-Sectional Study of 1055 University Students
Source: Nutrients. 2021 Oct 5;13(10):3512. doi: 10.3390/nu13103512 (PMC8537817; doi:10.3390/nu13103512)
Supplement: Supplementary file 1 [file nutrients-13-03512-s001.zip › nutrients-1381047-supplementary.pdf]

Factors related with diet quality. A cross-sectional study on 1055 university students.

Enrique Ramón-Arbués, José-Manuel Granada-López, Blanca Martínez-Abadía, Emmanuel Echániz-Serrano, Isabel Antón-Solanas, Benjamin Adam Jerue.

Supplementary Table S1. Criteria for scoring the Spanish Healthy Eating Index (SHEI).

Category	Criteria for a score of 10*	Criteria for a score of 7.5	Criteria for a score of 5	Criteria for a score of 2.5	Criteria for a score of 0
**Bread and grains**	Consumed daily	3 or more times a week but not daily	Once or twice a week	Less than once a week	Never or very rarely
**Vegetables**	Consumed daily	3 or more times a week but not daily	Once or twice a week	Less than once a week	Never or very rarely
**Fruits**	Consumed daily	3 or more times a week but not daily	Once or twice a week	Less than once a week	Never or very rarely
**Dairy products**	Consumed daily	3 or more times a week but not daily	Once or twice a week	Less than once a week	Never or very rarely
**Meat**	Once or twice a week	3 or more times a week but not daily	Less than once a week	Consumed daily	Never or very rarely
**Legumes**	Once or twice a week	3 or more times a week but not daily	Less than once a week	Consumed daily	Never or very rarely
**Cold meats and cuts**	Never or very rarely	Less than once a week	Once or twice a week	3 or more times a week but not daily	Consumed daily
**Sweets**	Never or very rarely	Less than once a week	Once or twice a week	3 or more times a week but not daily	Consumed daily
**Soft drinks with sugar**	Never or very rarely	Less than once a week	Once or twice a week	3 or more times a week but not daily	Consumed daily
**Variety of diet**	1 point if the participant completes each of the weekly recommendations and two points if the participant each of the daily recommendations.				

*A score of 10 in a particular category means that the participant's habits are aligned with the frequency recommendations for food consumption provided by the Spanish Society for Community Nutrition[1].

Reference

(1) Grupo Colaborativo de la Sociedad Española de Nutrición Comunitaria (SENC; Aranceta Bartrina, J.; Arija Val, V.; Maíz Aldalur, E.; Martínez de la Victoria Muñoz, E.; Ortega Anta, R. M.; Pérez-Rodrigo, C.; Quiles Izquierdo, J.; Rodríguez Martín, A.; Román Viñas, B.; Salvador Castell, G.; Tur Marí, J. A.; Varela Moreiras, G.; Serra Majem, L. Dietary guidelines for the Spanish population (SENC, December 2016); the new graphic icon of healthy nutrition. *Nutr. Hosp.* **2016**, *33* (Suppl 8), 1–48. https://doi.org/10.20960/nh.827.
